# Research and Remedies

**Published:** 1913-04-12

**Authors:** 


### RESEARCH AND REMEDIES.

### X-Rays for Uterine Fibroids.

Before the modern surgical treatment of uterine fibroid growths had attained the extraordinary success which has been a feature of the last fifteen years, all kinds of measures had been adopted for the relief of that very common and very troublesome malady. Electricity in various forms was advocated by some, but there is no doubt that it was a complete failure. Latterly the development of electro-therapeutics has induced further experiments on fibroids; and although many medical electricians are still very guarded in their pronouncements, there are some abroad who claim great achievements. Indeed, some of the enthusiasts are already speaking of the abolition of hysterotomy and its replacement by electro-therapy. At present these claims are undoubtedly exaggerated, and it is certainly true at present surgical operation holds the field. But it does not do to shut one's eyes to possible advances, and it is conceivable that the $x$-ray experts may some day evolve a treatment which will fulfil or partly fulfil their hopes. At Freiburg, Halle, Heidelberg, Tübingen, and at two or three clinics in France, systematic $x$-ray treatment is being tried: it is said that hæmorrhage can be stopped in two or three months, but the effect upon the tumour itself seems rather uncertain. The idea is to cause ovarian degeneration by exposure to the rays, with destruction of the corpora lutea and an artificial menopause. The tube recommended is a hard one, with a filter of glass or aluminium; and great care has to be taken lest $x$-ray burns of the abdomen should occur. An application once a fortnight, repeated three to six times, is said to be sufficient to arrest menorrhagia in most cases; and another advantage claimed is that the patient suffers from none of the acute symptoms of the menopause. For fuller information on the subject an abstract of the recent work abroad published in the *Edinburgh Medical Journal* should be consulted.

